# Why Do Patients and Caregivers Seek Answers From the Internet and Online Lung Specialists? A Qualitative Study

**DOI:** 10.2196/jmir.2842

**Published:** 2014-02-04

**Authors:** Romane Milia Schook, Cilia Linssen, Franz MNH Schramel, Jan Festen, Ernst Lammers, Egbert F Smit, Pieter E Postmus, Marjan J Westerman

**Affiliations:** ^1^VU University Medical CenterDepartment of Pulmonary DiseasesAmsterdamNetherlands; ^2^Lung Cancer Information Center: Longkanker InformatiecentrumMaarsbergenNetherlands; ^3^St Antonius Hospital NieuwegeinDepartment of Pulmonary DiseasesNieuwegeinNetherlands; ^4^Gelre ZiekenhuizenZutphenNetherlands; ^5^Vrije Universiteit AmsterdamDepartment of Health Sciences, Methodology and Applied BiostatisticsAmsterdamNetherlands

**Keywords:** lung cancer, patients, caregivers, website, online lung specialists, reasons, Internet, information needs, coping, qualitative

## Abstract

**Background:**

Since its launch in 2003, the Dutch Lung Cancer Information Center’s (DLIC) website has become increasingly popular. The most popular page of the website is the section “Ask the Physician”, where visitors can ask an online lung specialist questions anonymously and receive an answer quickly. Most questions were not only asked by lung cancer patients but also by their informal caregivers. Most questions concerned specific information about lung cancer.

**Objective:**

Our goal was to explore the reasons why lung cancer patients and caregivers search the Internet for information and ask online lung specialists questions on the DLIC’s interactive page, “Ask the Physician”, rather than consulting with their own specialist.

**Methods:**

This research consisted of a qualitative study with semistructured telephone interviews about medical information-seeking behavior (eg, information needs, reasons for querying online specialists). The sample comprised 5 lung cancer patients and 20 caregivers who posed a question on the interactive page of the DLIC website.

**Results:**

Respondents used the Internet and the DLIC website to look for lung cancer–related information (general/specific to their personal situation) and to cope with cancer. They tried to achieve a better understanding of the information given by their own specialist and wanted to be prepared for the treatment trajectory and disease course. This mode of information supply helped them cope and gave them emotional support. The interactive webpage was also used as a second opinion. The absence of face-to-face contact made respondents feel freer to ask for any kind of information. By being able to pose a question instantly and receiving a relatively quick reply from the online specialist to urgent questions, respondents felt an easing of their anxiety as they did not have to wait until the next consultation with their own specialist.

**Conclusions:**

The DLIC website with its interactive page is a valuable complementary mode of information supply and supportive care for lung cancer patients and caregivers.

## Introduction

Lung cancer is the second most common type of cancer and the most common cause of cancer deaths in both men and women in Europe and the United States [1,2]. The Netherlands counted approximately 14,000 lung cancer patients in 2002. This number increased to more than 21,000 in 2011 [3]. In 2003, the Dutch Lung Cancer Information Center (DLIC) was established. Its purpose was to give simple, accessible, and evidence-based information on lung cancer through its website [4], as well as support, and to unite lung cancer patients and their caregivers [5]. The unique quality of the DLIC is that it was set up at the national level and broadly supported by both health care professionals and patient groups. Following its creation, a special section was added to its website—an interactive webpage, called “Ask the physician”, where visitors could anonymously ask an online lung specialist questions and receive an answer within 48 hours [6]. It was after some scepticism from the lung specialists involved in the website’s management and content that this new section was launched [6].

Since its launch, the DLIC website has become increasingly popular and has reached a steady number of 25,000 unique visitors per month [5-7]. Surprisingly, the most popular page of the website is the “Ask the Physician” page. Our study group has previously investigated who was visiting the interactive webpage and what the information visitors were looking for [6,8]. Seventy-four percent of the questions (n=1893) were not only asked by lung cancer patients (13%) but also in large numbers by their informal caregivers (61%, eg, family, friends, and loved ones). Most questions (43%) concerned specific and general information about lung cancer. Furthermore, verification of information given by patients’ own specialists was sought, for example, the overall survival rate of lung cancer or specific therapeutic advice.

This impressive number of caregivers visiting the DLIC website, next to lung cancer patients, and their use of the online experts piqued our interest. Literature shows that a cancer diagnosis is an immediate reason for an increase in Internet use by patients and their families to obtain medical information, next to unmet information needs [9-13]. Looking for information seems to be an important and frequent task for caregivers, next to other activities, such as patient care, supporting and facilitating medical consultations, and aiding in information recall [14-16]. Also, physicians have limited consultation time and are not statutorily obliged to address or inform caregivers as they are patients, although such an approach is recommended [17,18]. Furthermore, consultations addressing multiple persons require high communication skills and are thus difficult. It is therefore plausible to think that unmet information needs underlie the above mentioned Internet use. However, if we look at the total picture, many elements and motives for these Internet searches remain unclear to us, especially with regard to caregivers of lung cancer patients in particular and the patients themselves. Why are there so many caregivers of lung cancer patients looking for information and consulting the DLIC online lung specialists? Why do they address the online specialists instead of the patient’s own specialist? Are there differences between caregivers and patients with regard to their motives when seeking information and their needs?

In comparison to other groups of patients, lung cancer patients and their caregivers are more vulnerable because the patients are facing a much shortened life expectancy. They need more special attention from health care providers. As the quality of life, psychological adjustment to the disease, risk of appraisal, anxiety, and depression of cancer patients and their caregivers are affected by barriers and failures in information supply and communication from health care providers [19-21], it is crucial that this vulnerable group receive information meeting their needs, especially because these needs differ throughout the cancer trajectory [22].

Adequate information supply is considered to be a part of good medical care and support. To provide appropriate care to lung cancer patients and their caregivers, it is important to explore their information-seeking behavior in order to gain more insight into their needs and indications for better communication modes and a tailored information supply.

Therefore, we conducted a qualitative interview study with telephone interviews to explore the reasons why caregivers and lung cancer patients search the Internet for information and ask the DLIC online lung specialists additional questions about lung cancer, next to face-to-face consultations with their own treating specialists.

## Methods

### Design

We conducted a qualitative, exploratory study consisting of semistructured telephone interviews with visitors to the DLIC website who asked the online lung specialist a question on the “Ask the Physician” webpage. This study was approved by the local medical ethics committee of the VU University Medical Center, Amsterdam.

### Procedures and Participants

Recruitment took place between August 2005 and April 2006. All consecutive visitors who asked the online lung specialists a question on the interactive page of the DLIC website were invited to participate in our study. After submission of a question, an online (digital) confirmation window would pop up with Dutch text, containing an invitation for participation in our study and an explanation about its purpose and the telephone interviews to be held. If visitors wished to participate, they were asked to complete an online form with their name, phone number, and home and email address in order to be contacted. After completion of the form, visitors could click on the button “send”. If they did not wish to participate, they could just close the pop-up window.

Within 3 weeks, visitors who had given their online consent for study participation were contacted by email or phone by CL (a communications expert and expert interviewer with no prior relationship to the study respondents). CL gave them additional information and checked whether participants were fluent Dutch speakers. When CL contacted the volunteers by phone and oral participation consent was given, they were either interviewed immediately, or a later appointment was made. If CL contacted them by email, written participation consent was given and an appointment was made for a future telephone interview.

Of the 84 persons who had agreed to participation online, 43 individuals could not be reached, 4 asked to postpone the interview but never contacted CL again, and 7 refused participation after initially having been interested (4 were due to the patient’s death/current poor condition, and 3 gave no reason). Ultimately, 30 participants were interviewed on their motives for looking for information on the Internet and asking questions on the DLIC “Ask the physician” webpage. Of the 30 participants, 5 were patients with a lung tumor, 20 were caregivers of lung cancer patients, and 5 did not have cancer (one lung patient and 4 individuals who feared that they had lung cancer).

Since we were interested only in cancer patients with a lung tumor and their caregivers, we analyzed only the 25 respondents with cancer. The ratio of patients and caregivers (5:20) is not balanced but is in accordance with the population of visitors of the interactive webpage, as we have reported in other papers [6,8]. One patient had small cell lung cancer (SCLC), three had non-small cell lung cancer (NSCLC), and one probably had breast cancer with lung metastases. The patients had a median age of 52 years (range 44-62). The majority of caregivers were women, most of them daughters and partners, with a median age of 39 years (range 21-58) (see Table 1 for more details on participants).

**Table 1:** Study population characteristics and Internet use (n=25).

	Gender M/F	Age, years	Diagnosis patient^b^	Previous Internet use^b^	Education^c^	Caregiver type	Current therapy
P^a^1	F	44	Metastasized BC	No	HE	–	Palliative therapy
P2	M	62	NSCLC I/II	Yes	HE	–	Adjuvant chemo
P3	F	50	SCLC ED	Yes	LE	–	Palliative chemo
P4	M	52	NSCLC SU	Yes	LE	–	Palliative chemo
P5	F	52	NSCLC I/II	Yes	HE	–	Adjuvant chemo
CG^a^1	F	57	NSCLC IV	Yes	HE	Partner	Palliative therapy
CG2	F	36	NSCLC IV	Yes	LE	Daughter	Deceased 6 weeks before
CG3	F	45	NSCLC IV	Yes	HE	Partner	Deceased 3 months before
CG4	F	52	NSCLC I/II	Yes	HE	Partner	After surgery, no adjuvant chemo
CG5	F	39	Mesothelioma	Yes	HE	Daughter	Terminal phase
CG6	F	39	SCLC LD	No	HE	Daughter	No current therapy, chemoradiation 1 year before
CG7	F	33	LC SU	Yes	LE	Daughter	Palliative chemo
CG8	F	51	NSCLC IV	Yes	LE	Partner	No current therapy, palliative chemo 6 months before
CG9	F	32	LC SU	Yes	LE	Daughter	After diagnostics and diagnosis
CG10	F	26	LC SU	Yes	HE	Niece	Deceased
CG11	M	22	Mesothelioma	Yes	LE	Nephew	Therapy unknown 6 months after diagnosis
CG12	F	42	LC IV	N/A	LE	Daughter	Palliative therapy
CG13	M	58	LC IV	Yes	LE	Partner	Palliative therapy (radiotherapy)
CG14	F	38	LC IV	Yes	LE	Sister	Palliative therapy
CG15	F	21	NSCLC I/II	Yes	HE	Daughter	No current therapy, surgery 1 year before
CG16	M	28	SCLC SU	Yes	HE	Son	Therapy unknown 3 months after diagnosis
CG17	M	36	LC IV	Yes	HE	Partner	Palliative chemo
CG18	M	35	LC IV	N/A	HE	Son	Palliative therapy
CG19	F	41	Mesothelioma	Yes	HE	Daughter	Deceased recently
CG20	F	44	LC SU/ metastases BC	Yes	LE	Daughter	Therapy unknown during diagnostics

^a^P=patient, CG=caregiver.

^b^BC=breast cancer, NSCLC=non–small cell lung cancer, IV=stage IV, SCLC=small cell lung cancer, ED=extensive disease, SU=stage unknown, I/II=stage I or II, LD=limited disease, LC=lung cancer type unknown.

^c^HE=high education (university, academy, college level), LE=low education (primary school, high school, intermediate vocational training).

### Interviews

A topic list was made to prepare the interviews. Main topics were Internet use, information needs and supply, reasons to use the Internet, reasons to query the online lung specialists on the DLIC website, and reasons not to. The topic list was completed with personal information about demographics and disease. Sample questions asked during the interviews are listed in Textbox 1.

The semistructured telephone interviews were conducted by CL. Participants were encouraged to talk freely until all topics were discussed. The duration range of an interview was 20-90 minutes. All interviews were written down verbatim with pen and paper, put into orthographic transcripts, and then subsequently typed into MS Word documents directly after interview termination (these MS Word documents will be referred to as “interview transcripts”). During transcription into MS Word documents, CL would already start to classify interview passages according to their content and the questions listed in Textbox 1 (topic list based). Apart from the interview transcripts, sometimes CL wrote notes with her impressions on the respondents’ ideas during the interviews, which she attached as a memo to the interview transcripts. Occasionally she also copied interesting quotes from her email correspondence with the participants as field notes into the interview transcripts.

**Sample interview questions.**

When did you start looking for information on the Internet?

Why did you look for information on the Internet?

What role does the Internet play in information supply?

What role does the caregiver play in information supply?

Why did you ask a question on the interactive page of the DLIC website?

What did you ask? What did you want to know?

Was the answer to your question satisfactory? Was it useful? Why?

At which moment during the lung cancer procedure did you have the greatest information needs?

Why did you not ask the (patient’s) treating physician your question?

Is it different to ask a question through the Internet/by email? Why? How so?

What is your opinion about the possibility of asking an online physician questions on a website?

What is your opinion about the possibility of asking your treating physician questions by email?

What is your opinion about the possibility of asking a nurse questions by email?

Would you like to communicate with the (patient’s) treating physician by email?

What would be the value of such communication?

### Analysis

Researchers RMS and MJW used a thematic approach in the analysis of the transcripts (n=25) [23]. After familiarization with the data by reading it repeatedly and carefully, we made a summary of each interview and started initial coding of the transcripts. To facilitate coding, organizing, collecting, and selecting data from the interview transcripts, we used MaxQDA version 10 [24]. After numerous meetings focusing on understanding the collected data and correct interpretation, we determined potential themes first and then sorted and collated the (initial) codes according to them. Hence, we looked at the participant’s Internet use first, to further determine when they started to surf the Internet and to assess their information needs. After this, we focused on identifying and classifying the reasons why participants surfed the Internet and posed questions to online lung specialists instead of their own specialist. Grouping this information, we made an initial thematic map (available on request).

After review of the potential themes for coherence, we refined these themes, identified new themes, and recoded some data extracts. This refinement led to the identification of similarities and discrepancies between participants with regards to the sought-after information. The newly identified themes were found to be the beneficial effects of looking for information for participants, the presence of tension between patients and caregivers provoked by the Internet search, and perspectives about the use of email with the patient’s specialist. After recoding the data extracts according to the refined and new themes, we reviewed the entire dataset again and discussed the generated main themes conscientiously and critically for coherence, consistency, robustness, and representativeness [23,25] in order to develop a final thematic map (available on request).

## Results

### Starting to Surf the Internet

All respondents, except for 1 patient and 3 caregivers, reported using the Internet on a regular basis. They had access to Internet at home and used it for daily activities such as checking their email, banking, or looking for different types of information. They reported that the lung cancer diagnosis specifically urged them to seek information and ask the online lung experts lung cancer–related questions. Their diagnosis had a great impact on their lives, as they were facing lung cancer, its (future) treatment trajectory, and ultimately the shortened life expectancy of the patient. Therefore, they felt the need for additional information to learn how to deal with the situation by any means.

Both patients and caregivers also mentioned that they surfed the Internet again at specific moments later during the lung cancer treatment trajectory, such as during chemotherapy, at the appearance of new symptoms or disease progression, or when having to make a choice between two treatment options. These moments also meant a change in their current vulnerable balance, which pushed them to search for information again.

I have been told a lot at the hospital, but everything goes so fast, you hear a lot of terms, and you just do not know anything […] First, I looked at the tumor types and how everything would go during surgery. After that, I looked again when it was recommended for me to have chemotherapy.

P2

Patients and caregivers mentioned that their need to seek information often arose once they had time to rest and think about what they had been told, often at a time when their questions could not directly be answered by the treating specialist anymore: “Once you have come home, you have forgotten half of what you have been told, which is exactly the moment you would want to ask something.” [CG8, partner]

### What Are Respondents Looking For and Why?

Respondents reported searching for lung cancer–related information in general but also information specific to their personal situation. An illustration of the information search of caregiver Sylvia (fictitious name), describing what she was looking for and why, is given in Textbox 2.

Apart from feeling the need to gather general information in order to be better informed and have a better understanding of the disease, respondents wanted to be prepared for future consultations, future course of disease, and treatment trajectory. They also felt the need for specific information regarding practical matters or emotional support directly related to their individual condition in order to help face current or short-term situations. Examples are practical matters during the treatment trajectory and finding emotional support through contact with fellow sufferers (see Table 2). Ultimately, all respondents expressed that the main goal of their information search was to find support as they were dealing with lung cancer. Textbox 3 also gives an illustration of patient Mary’s (fictive name) information search and her search motives.

**Caregiver Sylvia.**

Sylvia (fictitious name) is the 36-year-old daughter of a lower educated male patient of 72 years old. At time of the interview, Sylvia’s father had died a week earlier. He had been diagnosed with metastasized lung cancer 7 weeks prior.

I started looking [for information] 2 weeks after the definitive diagnosis of lung cancer with brain metastases had been made. I searched the Internet because I wanted to know the prognosis and what different types of lung cancer there were. Once I started, I kept on going. I also wrote something on the forum of the DLIC website and I got some reactions; it was very nice. It may sound strange, but it is nice to know that there are many people who are dealing with the same thing. At the hospital they don’t have much time for you. You can see them thinking “Yes, you have cancer, I have explained everything, now get on with it”. Then you come home and the questions arise […] and you think “I want to ask the question now”. But if you call the doctor, you get the secretary who says “the doctor is not here, he is with a patient. When do you have an appointment? Next week? You can ask your questions then”. But this way a question that feels urgent to you remains unanswered. This is one of the reasons why I turned to the Internet and by stumbling across the DLIC website, […] I found everything I was looking for. This website is incredible. I can stay on the site for hours […] When I came back to the treating physician after the diagnosis, I asked him “what kind of lung cancer is it, small cell, non–small cell? What are the advantages and disadvantages of giving therapy?” You should have seen his face wondering how I knew all of that. Actually, I have only ever asked the online expert one question: “if someone has metastasized cancer, why is not it possible to operate on the lungs and brain and just remove the cancer from both sites?” I received a satisfactory answer. Although it was just as I thought, it was still nice to get confirmation. And you never know, perhaps the Internet expert will say there are still possibilities or new therapies. Even if it is not the case, it is still nice to have been able to ask. I think it is excellent to be able to ask a question of the DLIC online expert and to get an answer so quickly because it has prevented several sleepless nights.

**Patient Mary.**

Mary (fictitious name) was a 44-year-old highly educated, married patient. She was diagnosed with breast cancer metastasized to the lungs. After a period of stable regression, she was receiving palliative therapy at time of the interview. She was very pleased with Internet as mode of information supply and the DLIC interactive webpage, but she emphasized that she did not want eHealth to become a substitute for visual contact with treating specialists in the future.

After the diagnosis, I hit rock bottom. A neighbor, who is a nurse, brought me a lot of pamphlets. This helped me back on top and gave me the feeling that I should do something. At that point [1 month after the diagnosis], I started to study the folders and the Internet. I wanted to come to grips with the situation and also get the feeling that I had a rough plan for my treatment. The Internet has played an important role in terms of information supply. I am a member of a private mailing group where we exchange a lot of information. One of the group members made me aware of the DLIC website and that questions were being answered there. Early on, I would see my doctor first and after that I would go online looking for the things he had suggested, verifying whether there were no other possibilities. But there came a point when I felt I needed to take charge of the situation instead of just following him passively. I wanted to get ahead of the game, so now I started looking for information before every new hospital appointment, so that I could come well prepared. I researched every possible thing: what I had, what I would get, what possible alternative or experimental therapies there were, things about pain control for later during the process, you name it. I have accepted the disease and I see myself as a kind of research project. I think it is good to be well informed. It gives you the feeling that you have control over your disease and your treatment.

**Table 2:** Needs of respondents.

Information needs		Examples
**General information needs**		
	Background information on lung cancer	Epidemiology, lung cancer types
	Diagnosis	
	Therapy	Regular, experimental, alternative
	Diagnostics	Investigations types and explanation about it
	Disease course/end of life/prognosis	Life expectancy, (overall) survival per stage, what to expect at the end of life, suffocation
	Information sources/literature	DLIC website, websites specialized on experimental therapy
**Individual information needs**		
	Managing personal situation	Help with a choice: postoperative chemotherapy, radiotherapy or not
	Managing personal health or mental condition	Explanation and/or treatment of symptoms or side effects (eg, own neurological problems or insensibility after surgery), preparation for coming treatment (eg, what is going to happen during surgery), analgesia, what can this symptom be?
	Managing emotions	Search for hope, confirmation, reassurance, emotional support, compassion, consolation, contact with fellow sufferers/ comparable experiences, expert’s verification/2^nd^opinion, moments of panics and uncertainty
	Managing daily life	Lifestyle advices, hospital bills, food supplements, hospice
	Managing practical aspects of treatment trajectory	Eg, explanation of medical terms/terminology like stable disease or “adenocarcinoma”, meaning of laboratory/imaging results specific to patient, organizing euthanasia

### Differences Between Patients and Caregivers

Although patients and caregivers reported searching for general information as well as information specific to the individual condition, their searches differed with regard to quantity and content (see Textboxes 2 and 3). Patients searched for a minimal amount of general background information and focused specifically on their individual current condition. They aimed to get more information about symptoms and therapy, together with practical information for the coming disease course and consultations. Not all of the patients seemed to want to gather information about the last moments of life, although they were perfectly aware of their shortened life expectancy. Patients especially said they were searching (among other things) for hope by contacting fellow sufferers and/or looking for a confirmation of the accuracy of their diagnosis and chosen management. This made them feel supported (see Textbox 3).

On the contrary, caregivers expressed the need to collect a lot of general information of any kind (see Textbox 2). An element they mentioned with regard to the available information on the Internet was the difficulty of understanding or interpreting online information correctly, as they were lacking a doctor’s knowledge and felt overwhelmed by the vast amount of information given. Caregivers also wanted to be informed more frequently about the patients’ end of life and prognosis in particular. Furthermore, they reported looking for information specific to their personal situation in order to feel supported. Like the patients, caregivers said that their quest for (emotional) support consisted of (among others) searching for hope and reassurance/confirmation of medical information, contact with other fellow sufferers (by direct contact or by reading their stories) and with the online expert (see Table 2).

### Why Not Ask Their Own Treating Specialist?

Numerous reasons were mentioned for using the Internet and asking the online expert questions in particular, rather than addressing their own treating specialist. Respondents said they did not *want* to ask their own specialist. They mentioned being ashamed about discussing personal matters or indecencies within the context of a consultation. They also did not wish to disturb or burden their own specialist because he or she was (supposed to be) very busy. Especially caregivers were convinced of being a burden to the specialist as they were “only a caregiver anyway”.

There are a lot of things that I wouldn’t discuss with the lung specialist. For example, a big part of his lung has been removed and when we caress each other, he has no feeling in that part of his skin. He does not feel my touch. He says that it feels alien, as though it’s not part of his own body. Then we asked ourselves “will it remain that way?” But you do not ask the lung specialist those kind of things when he is looking at the chest X-ray very seriously.

CG4, partner

And you do not bother the specialist by calling him at the hospital, because surely he has better things to do.

P3

Respondents had the feeling of not being able to ask a question or request information. They experienced barriers in their contact with their own specialist because they felt he or she had no time during and between consultations. They experienced the specialist not being open to questions: “the communication at the hospital was dramatically bad” and “We have a kind of reticence to ask our treating specialist questions. They do not like it when you ask a question.” [CG6, child]

Following the suggestion of the DLIC, I brought a list of questions for my specialist. You could see him thinking “Not another one with a list…” Specialists are always in a hurry. They do not even have time for a proper discussion with you. I have seen 4 different pulmonologists, and when you walk into the consultation room, they would all still be reading your medical record at the same time.

P2

Patients as well as caregivers appreciated the convenience of using the Internet and the DLIC website because of its 24-hour accessibility and its anonymity. This made them feel freer to ask the online lung experts for any kind of information and helped them express their feelings better. They felt less anxiety as they were able to pose a question instantly, receive a reply from the online specialist to urgent questions within a short space of time, and not have to wait until the next consultation with their treating specialist. This was especially the case for caregivers as, for example, one of them said that the patient had a follow-up appointment every 3 months but that she could surf the Internet every day. Furthermore, respondents appreciated the expertise and open-minded, kind, and empathic attitude of the online lung experts.

I think it’s fantastic. It is anonymous and it’s great to ask your questions to someone who’s competent in the field […] I was looking for someone independent […] Although he [the online expert] is an outsider, he knows what he is talking about.

P2

I think it is very good to be able to ask a physician questions online. It’s a smaller step to take than calling or talking to your treating specialist.

CG9, child

His style (of the online expert) is really nice, not disapproving. He is very kind and always says something like “I wish you good luck” or “I hope it will be all right”.

CG1

### Reasons Not to Use the Internet

Patients and caregivers mentioned that sometimes they postponed or stopped their Internet search, for instance, because the information they encountered was too much. Not searching helped them to stay positive. Other respondents felt that they had collected enough information after a period of time and therefore deliberately quit the information search, knowing they were avoiding confrontation with the disease sometimes.

No, at the beginning, I did not look for information. The disease, it was not about me, it was as if it was about someone else […] I must say that I am not on the Internet very often anymore, because a lot of people die there. It is too much for me [...] and makes me feel depressed. You need to feel there is still a light at the end of the tunnel.

P3

I went looking for information after my husband passed away, not during his illness though. It does not make you feel happy and I wanted to stay positive, so it does not help if you read these unpleasant things on the Internet.

CG3, partner

I think I should not read too much about lung cancer anymore. Now that the disease has gone, it’s time to move on. I have got the feeling that I have just recovered from a heavy illness myself and that at long last I am finally fit enough to get up and go again. Yes, perhaps I am avoiding thinking about all that is lost.

P2

### Tensions

Both patients and caregivers also talked about the occurrence of tension when meeting their own information needs by searching the Internet. Specifically caregivers realized that their needs were not always the same as the patients’ and experienced difficulties in dealing with the information they had collected. They felt torn by the dilemma of disclosing sensitive information or hiding it from the patients, as they wanted to protect them from (unwelcome) confrontations. For example, one caregiver said that he did not share the death of someone from his mailing group as he thought that this would be too much to handle for the patient.

There are things that I do not tell him, because I do not want to worry him […] It is difficult because sometimes, when we are at the doctor’s, I would like to know things, such as the life expectancy, but I am reluctant to ask, because I do not want my father to hear it.

CG12

Well…Actually I have kept information from him when he was very unwell and we did not know yet whether he could be operated. At the time, my son and I looked for the 5-year survival rate and decided we should not tell him. Once he was home again after the operation, he was looking at a very old medical encyclopedia that we never use. Then he said “Do you know what the 5 years survival of lung cancer is?” I said I did and in reply he asked me why I had never told him. He was upset at first, but he understood.

CG4, partner

### Email Contact With Their Own Treating Specialist or an Oncology Nurse

All patients were very positive when being explicitly asked about their opinion on the opportunity to have email contact with their own specialist for questions. Caregivers, however, had more reservations as they felt embarrassed contacting the treating specialist (as being *only* caregivers) and were afraid that the specialists might be overwhelmed by emails.

Respondents also reacted positively about having email contact with an oncology nurse in order to obtain medical information and ask questions, on the condition that she or he had to specialize in lung cancer. One caregiver mentioned the very useful assistance of an oncology nurse as a constant and accessible contact point for support and information during the whole treatment trajectory.

## Discussion

### Principal Findings

The present study adds knowledge to the information-seeking behavior of lung cancer patients and their caregivers during the lung cancer treatment trajectory and their reasons for doing so. Strikingly, the majority of respondents were caregivers. Our findings show the coping strategies of caregivers and patients towards managing lung cancer. They searched the Internet and asked online DLIC lung experts questions because they wanted lung cancer-related information and help in coping with the disease practically and emotionally. This happened repeatedly during the whole treatment trajectory. This search helped the respondents to deal with lung cancer in a better way. It permitted them to gain a better understanding, be prepared (for the treatment trajectory and the disease course), feel free to search and ask for information, express feelings, be relieved of anxiety, feel emotionally supported, and regain control. This confirms that information is essential and beneficial for coping with cancer for both patients and caregivers and that caregivers are actively involved in information search and supply [14,26]. Furthermore, the information needs of caregivers differed from those of patients.

Lung cancer patients and their caregivers searched the Internet in order to deal with lung cancer and their personal situation. The perspective of the coping theory can be applied to explain the respondents’ behavior [27]. When events occur in a subject’s life, the subject is prompted to activate internal processes necessary to accommodate that event (eg, behavioral, cognitive, and affective mechanisms, including coping). It is known that anxiety, anger, fear, helplessness, and depressive feelings are frequently experienced after a cancer diagnosis [28]. The study respondents experienced such distress at diagnosis and other key points of the treatment trajectory that provoked a change in their lives, therefore posing a threat, challenge, or harm to them personally. Subsequently they tried to manage this distress by means of coping, through searching the Internet and turning to online lung experts for lung cancer-related information.

Information is essential for coping with cancer, and new media (eg, the Internet, online cancer communities, mailing groups, etc) are crucial today for the dissemination of information [14,26]. Hence, our respondents are used to searching new media for information. Still, the choice of the Internet and online experts versus the patient’s treating specialist remains intriguing. Caregivers particularly had a greater tendency to search the Internet. This may be related to the serious nature of lung cancer, as the gravity of a disease urges people to seek additional information [14]. Also, when facing a life-threatening disease, cancer patients and family members often want confirmation of information, despite good communication with health care providers and adequate information supply [29,30]. Furthermore, Ong et al [31] and other investigators [32-35] found that patients and caregivers are often unsatisfied with the communication or the information given to them in medical settings. These issues were also observed in present study results. Apart from this, the practical advantages of the Internet and the availability and attitude of the online experts moved the respondents toward this medium.

Respondents were not only looking for general lung cancer-related information but also information specific to their own situation. Soothill et al [36,37] reported the need for “universal” and “personal” information among cancer patients and caregivers, helping them to cope with cancer, such as managing daily life or emotions. Searching for these two types of information was beneficial for the study respondents. Although similarities in the information search of patients and caregivers were observed, important differences were noticed too. Caregivers were inclined to look more extensively (in terms of quantity) for information than patients, and the content of the information found differed too. This trend was recognizable from the literature. Caregivers, of lung cancer patients in particular, show high participation rates in online cancer communities [14]. Compared to patients, they also have a higher tendency to look for information than providing it to other caregivers and patients, and they are more inclined to participate in emotional support exchange [6,8,14]. Moreover, lung cancer patients and caregivers have different information needs [38]; caregivers tend to have more unmet needs and concerns than patients [36,37]. This could originate from the caregivers’ perception of themselves as being helpless observers, their lesser involvement with health care providers, or the patients’ underreporting of concerns and unmet needs [39]. It also seems that information seeking is a typical activity for caregivers, as lung cancer patients are often too ill to do it themselves [14]. Interestingly, most caregivers among our respondents were (young) women. Women typically participate more in mail groups and supportive communication than men and seem to search or care more about information (provision) than men [14,40,41].

Thus, it is important to recognize the caregivers’ needs as well as those of patient, since caregivers play a critical role in sustaining the cancer patient, and their ability to nurture and support the patient may be compromised in case of unmet needs [42,43]. This may have serious implications for both the patients’ and caregivers’ psychological state and coping. Further investigations on this topic are therefore needed.

As seen in our results, trying to meet one’s own information needs can also be accompanied by difficulties and/or tensions. Confrontation with threatening or negative disease information can be of great impact on the well-being of patients and caregivers [11,44]. This may subsequently lead to the total abortion of the information search, the avoidance of confrontation with the “sensitive” information in particular, or concealing it from loved ones or patients, with all its possible consequences on the psychological state of those involved. This dilemma between wanting to meet information needs and protecting oneself or another have often been described in literature as the origin of conflicts and communication problems between caregivers and patients [45-47]. A balance between these two elements must therefore be achieved to maintain psychological well-being. Solutions to reach such a balance may not only be ceasing the information search temporarily, but also consulting reliable and clearly categorized sources of information and discussing the tension with the concerned persons or with someone who might be of help. The possibility of obtaining or discussing information directly with the treating physician or a specialized oncology nurse should be considered.

### Study Limitations

Since we performed a cross-sectional study with an interview at one single point during the lung cancer treatment trajectory, it is possible that we have missed information on the respondents’ needs, as we did not follow them over time. Nonetheless, respondents described different moments during lung cancer treatment. The quality of a person’s information needs is constant over time even though the quantity of the needed information may show a slight decrease [48,49]. It is therefore reasonable to assume that our results paint a reliable picture of the information needs and other reasons why respondents surfed the Internet.

Another limitation is the fact that all interviews were held by phone and were not audio recorded. Also, CL started to classify interview passages shortly after their transcription. This may have led to bias and to information loss during the simultaneous transcription of the interviews. However, since CL is an expert interviewer used to collecting information in this particular manner, it seems less probable that data were lost. Furthermore, the classification of interview passages took place according to the questions CL asked during the interviews (see Textbox 1), reducing bias.

Because data collection took place a few years ago, changes in habits of Internet users and DLIC website visitors may have occurred over time, next to changes in website availabilities. The relevance of our findings may also be questionable. However, we know that Internet health searches have become much more commonplace [50]. Additionally, internal reports of the DLIC website have shown that the number of visitors each month and the visitors’ identity remained the same over time [7]. The interactive webpage still remains a very popular page of the website, and the number of questions is still increasing. Questions concerning general information on lung cancer as well as information on personal matters are still being asked. The website availabilities have not really changed, and its homepage shows only a few additions since its launch (eg, animation, links to new blogs, and a visitor’s poll). We can therefore assume that the reported findings are still relevant and representative for the population we investigated.

A final question to address is whether our study sample is representative of the investigated population, as ultimately a sample of 25 respondents was interviewed despite the far larger number of persons interested. Persons who never surf the Internet were also anticipatorily excluded. The respondents’ distribution is, however, in accordance with the population visiting the DLIC website [6,8]. Moreover, patients and caregivers who never surf the Internet were not the target of this present study. Additionally, we showed in previous studies that many website visitors only read the webpage “Ask the physician” without asking questions. This group of visitors may be represented by the group of respondents who were interested in participating but were never interviewed. Another argument for the respondents’ representativeness is the reaching of saturation of data and themes after multiple readings of the interviews.

### Practical Implications

Our study results have numerous practical implications for the care of patients and their caregivers during the lung cancer treatment trajectory. Caregivers represented the majority of respondents looking for information and indicated they needed help coping with lung cancer. However, they often felt unable to address the patient’s treating specialist. Since the well-being of patients and caregivers are connected, special emphasis must be given to the often neglected experience and needs of caregivers [51,52]. In practice, this might simply be solved by addressing caregivers’ needs during consultations. In case of difficulties, lack of time, or objections from the treating specialists, workshops directed towards communicating with multiple persons and managing consultation time might help. Moreover, appointments additional to regular consultations are possible, as well as the implementation of support groups and information events focusing on the patient-caregiver unit. Extensive research on these possible interventions should be done prior to any implementation. The experiences, needs, and the role of caregivers during lung cancer treatment require further investigation.

Both patients and caregivers searched the Internet and the DLIC website broadly for additional information on lung cancer. It can therefore be concluded that there is a demand for such a service, although it was not considered a potential replacement for live consultations with the treating specialists. The positive effects on the respondents’ coping and their level of satisfaction, however, show that use of such services is favorable. They should therefore be promoted as *additional* information supply sources and be part of good medical care. To prevent the use of unreliable information sources, treating specialists might refer patients and caregivers to reliable and objective websites (with online experts). James et al have already reported evidence supporting this approach as being (surprisingly) a wish of both patients and caregivers [53].

Next to referral to a specialized oncology nurse for additional information and support, the development of direct personal email contact with the nurse or with the treating specialist may also be considered, for those reluctant to use the Internet and consult online experts. There are, of course, barriers and advantages to such communication modes [54]. Barriers might be the lack of Internet access and peer pressure, as well as the absence of training or ability to use email and concerns about junk mail, privacy, and security [54]. Advantages are numerous, such as speed, efficiency, and productivity [54], and, as illustrated in our study results, satisfaction and relief of anxiety among patients and caregivers. The specialists’ and nurses’ perceptions and the feasibility of direct email contact should nevertheless first be investigated before future implementation.

### Conclusions

Lung cancer patients and especially their caregivers use the Internet and the interactive webpage of the DLIC website because they want additional information on top of what they have received from their treating specialists. The information search also helps them to cope with lung cancer. The Internet and the DLIC’s interactive page are therefore valuable complementary modes of information supply. Because the DLIC online expert is not able to answer patient-specific questions, using email contact between patients/caregivers and treating specialists or specialized oncology nurses might be considered in case of urgent questions, next to referring them to reliable sources of information.

## Abbreviations

DLIC: Dutch Lung Cancer Information Center
NSCLC: non–small cell lung cancer
SCLC: small cell lung cancer
